# The Impact of Alkaliphilic Biofilm Formation on the Release and Retention of Carbon Isotopes from Nuclear Reactor Graphite

**DOI:** 10.1038/s41598-018-22833-5

**Published:** 2018-03-13

**Authors:** S. P. Rout, L. Payne, S. Walker, T Scott, P. Heard, H. Eccles, G. Bond, P. Shah, P. Bills, B. R. Jackson, S. A. Boxall, A. P. Laws, C. Charles, S. J. Williams, P. N. Humphreys

**Affiliations:** 1 Department of Biological Sciences, School of Applied Sciences, University of Huddersfield, Queensgate, Huddersfield HD1 3DH UK; 2 Interface Analysis Centre, University of Bristol, Tyndall Avenue, Bristol BS8 1TL UK; 3 Centre for Materials Science, University of Central Lancashire, Preston, PR1 2HE UK; 4 John Tyndall Institute for Nuclear Research, School of Computing, Engineering and Physical Sciences, University of Central Lancashire, Preston, PR1 2HE UK; 5 The Centre for Precision Technologies, University of Huddersfield, Huddersfield, HD1 3DH UK; 6 Bio-imaging Facility, School of Molecular and Cellular Biology, Faculty of Biological Sciences, University of Leeds, Leeds, LS2 9JT UK; 7 Department of Chemical Sciences, School of Applied Sciences, University of Huddersfield, Queensgate, Huddersfield HD1 3DH UK; 8 Radioactive Waste Management, B587, Curie Avenue, Harwell, Oxford OX11 0RH UK

## Abstract

^14^C is an important consideration within safety assessments for proposed geological disposal facilities for radioactive wastes, since it is capable of re-entering the biosphere through the generation of ^14^C bearing gases. The irradiation of graphite moderators in the UK gas-cooled nuclear power stations has led to the generation of a significant volume of ^14^C-containing intermediate level wastes. Some of this ^14^C is present as a carbonaceous deposit on channel wall surfaces. Within this study, the potential of biofilm growth upon irradiated and ^13^C doped graphite at alkaline pH was investigated. Complex biofilms were established on both active and simulant samples. High throughput sequencing showed the biofilms to be dominated by *Alcaligenes sp* at pH 9.5 and *Dietzia* sp at pH 11.0. Surface characterisation revealed that the biofilms were limited to growth upon the graphite surface with no penetration of the deeper porosity. Biofilm formation resulted in the generation of a low porosity surface layer without the removal or modification of the surface deposits or the release of the associated ^14^C/^13^C. Our results indicated that biofilm formation upon irradiated graphite is likely to occur at the pH values studied, without any additional release of the associated ^14^C.

## Introduction

The historical use of graphite moderated, gas cooled nuclear reactors by the United Kingdom is expected to generate 59,000 m^3^ of irradiated core graphite^1^ during decommissioning, this graphite is estimated to contain 7000 TBq of ^14^C^2^. In some cases reactor operations have led to the formation of a carbonaceous deposit on exposed graphite surfaces^3^ which has been shown to be enriched in ^14^C^4^. ^14^C is an important radionuclide for consideration in the safety assessment of a geological disposal facility (GDF) partly due to the radiological impacts of gaseous ^14^C species^5^. A number of the illustrative disposal concepts for the UK’s Intermediate Level Wastes (ILW) including ^14^C bearing wastes, are under consideration. Some of these concepts involve the use of a cementitious backfill material^5^ which along with corrosion processes will contribute to the generation of an anoxic, alkaline environment post closure^6^.

Evaluations of the radiological impacts of gaseous ^14^C species generally consider ^14^C labelled methane to have the greatest impact due to its mobility and the precipitation of ^14^C labelled carbon dioxide in alkaline systems^5,7,8^. This generation generally has a microbial component^9^ either through the direct transformation of ^14^C bearing compounds or through the metabolism of other gases that can influence the transport of ^14^C containing gaseous compounds^5,10^. Recent studies have found that about 0.07% of the ^14^C inventory present in Oldbury Nuclear Power Station reactor graphite may be abiotically released into the aqueous phase within 1 year, with 1% of this ^14^C being released to the gaseous phase on leaching in highly alkaline water (pH 13.0)^11^. The release of ^14^C in these experiments was a two phase process with an initial more rapid release seen in the first 28 days.

In addition to reactor graphite; the UK ILW inventory also includes an estimated 2,000 tonnes of cellulosic materials^12^ which under the anoxic, alkaline conditions anticipated to occur in a cementitious GDF will be subject to abiotic, alkaline hydrolysis^13^. This chemical hydrolysis process generates a range of soluble cellulose degradation products (CDP) dominated by isosaccharinic acids (ISAs)^14^. Previous studies have shown that CDP can support complex alkaliphilic, methanogenic, microbial communities^15–17^.

The disposal of irradiated graphite intermediate–level waste packages with other ILW packages that could contain cellulosic wastes may allow CDP to support the microbial colonisation of graphite surfaces. Graphite associated biofilms are synonymous with microbial fuel cells, with biofilms being observed on graphite surfaces in the presence of cellulose^18^. Studies involving biofilm formation using CDP as a carbon source are limited. Initial studies by Grant *et al*. showed that microbial communities isolated from alkaline Crater Lake were capable of forming biofilms on plastics and cementitious materials with an ISA substrate^19^. More recently, the work of Charles *et al*. showed that emplaced cotton cellulose within a highly alkaline site provided not only a substrate for CDP generation, but also a surface on which biofilm could form, allowing a flocculate-forming CDP-degrading community to be sub-cultured^16^. These flocs have been shown to provide protection against alkaline pH values by creating internal low pH environments^20^ and be able to facilitate biofilm formation on cementitious backfill material and a range of surfaces relevant to potential UK ILW disposal concepts^21^.

Since a proportion of the ^14^C present in some reactor graphite is associated with surface deposits^4^, microbial biofilms could influence the release of ^14^C from the graphite surface. The radiological activity of ^14^C limits its ease of manipulation in the laboratory, however recent advances have led to the development of a ^13^C labelled deposit. These deposits have been shown to be analogous to the ^14^C deposits observed on exposed reactor graphite surfaces in terms of topology and internal morphology^22,23^. These simulants allow the microbial impact on the release of isotopic carbon to be evaluated under alkaline conditions. The aim of this study was to determine the impact of alkaliphilic biofilm formation on the surface of ^13^C doped graphite and irradiated reactor graphite containing ^14^C, on the release and retention of these carbon isotopes.

## Results

### Irradiated graphite microcosms

**Figure 1:**
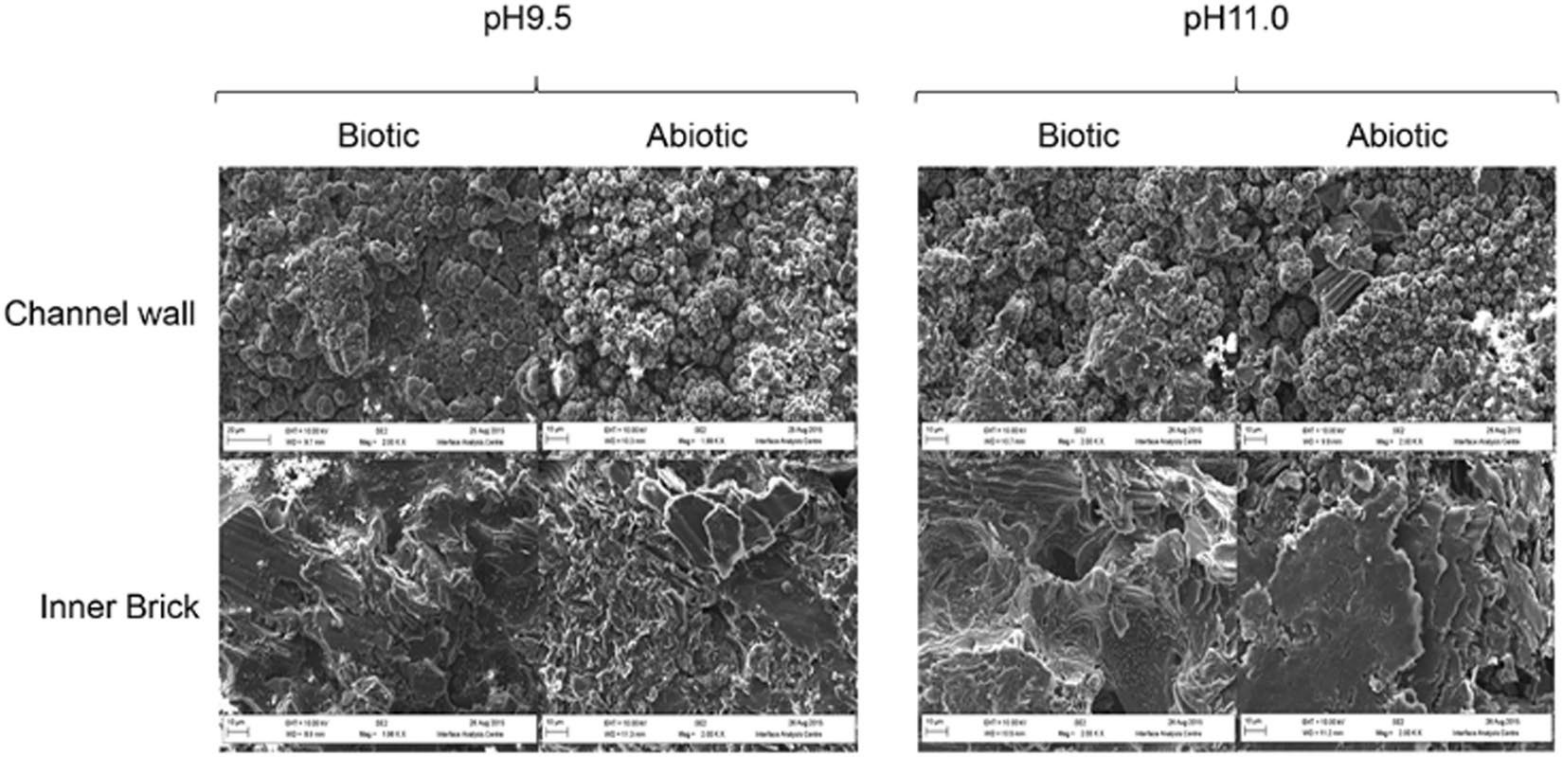
SEM imaging of active graphite samples.

Biofilms were established on the surface of the irradiated graphite samples within 12 weeks of incubation and could be observed via macroscopic investigation. Subsequent SEM imaging showed that a biofilm had formed upon the surface of the biotic microcosms containing either inner brick or channel wall samples at both pH values (Fig. 1). Within the abiotic channel wall samples the cauliflower-like topology (described in detail in Payne *et al*.^22^ and Payne *et al*.^23^), of ^14^C deposits could still be seen, with a clear biofilm coating the surface of these deposits observed on the biotic samples. In a similar fashion, clusters of cells and biofilm could be observed on the surface of the inner brick samples. Initial headspace analysis using RGA could not detect any clear ^14^C bearing gases in either the biotic or abiotic experiments indicting that there was no evidence that microbial action enhanced ^14^C release. In a similar fashion, LSC of the liquid phase and headspace analysis showed no discernible difference across samples, both biotic and abiotic (Fig. S2). In all cases, the activity detected in these samples was not significantly greater than those observed within the negative controls. This suggests that the amount of ^14^C mobilised is below the 4.3 Bq/mL detection limits of the investigation. Given that the average ^14^C content of Oldbury graphite is 84 KBq/g^11^ then 0.5% of the available ^14^C would have to be mobilised to be detected in these experiments. Due to the radioactivity of these samples it was not possible to carry out CLSM investigations.

### Simulant graphite microcosms

**Figure 2:**
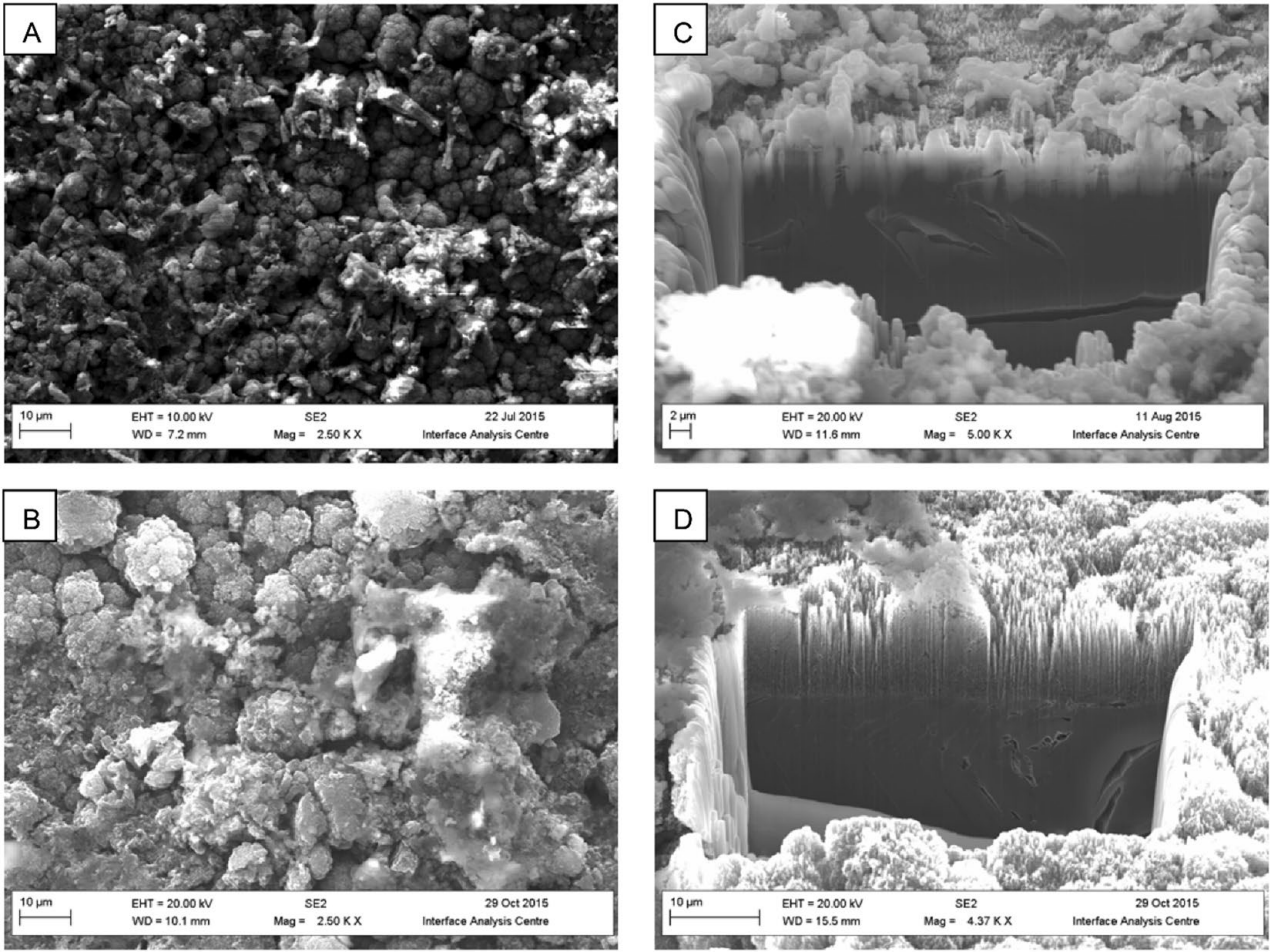
SEM/FIB-SEM investigations of C-13 simulant. Surface topology of biofilms present on C-13 simulant grown at pH 9.5 (**A**) and pH 11.0 (**B**), internal changes to morphology were then examined using FIB-SEM where the pH 9.5 (**C**) and pH 11.0 (**D**) associated biofilms are shown.

**Figure 3:**
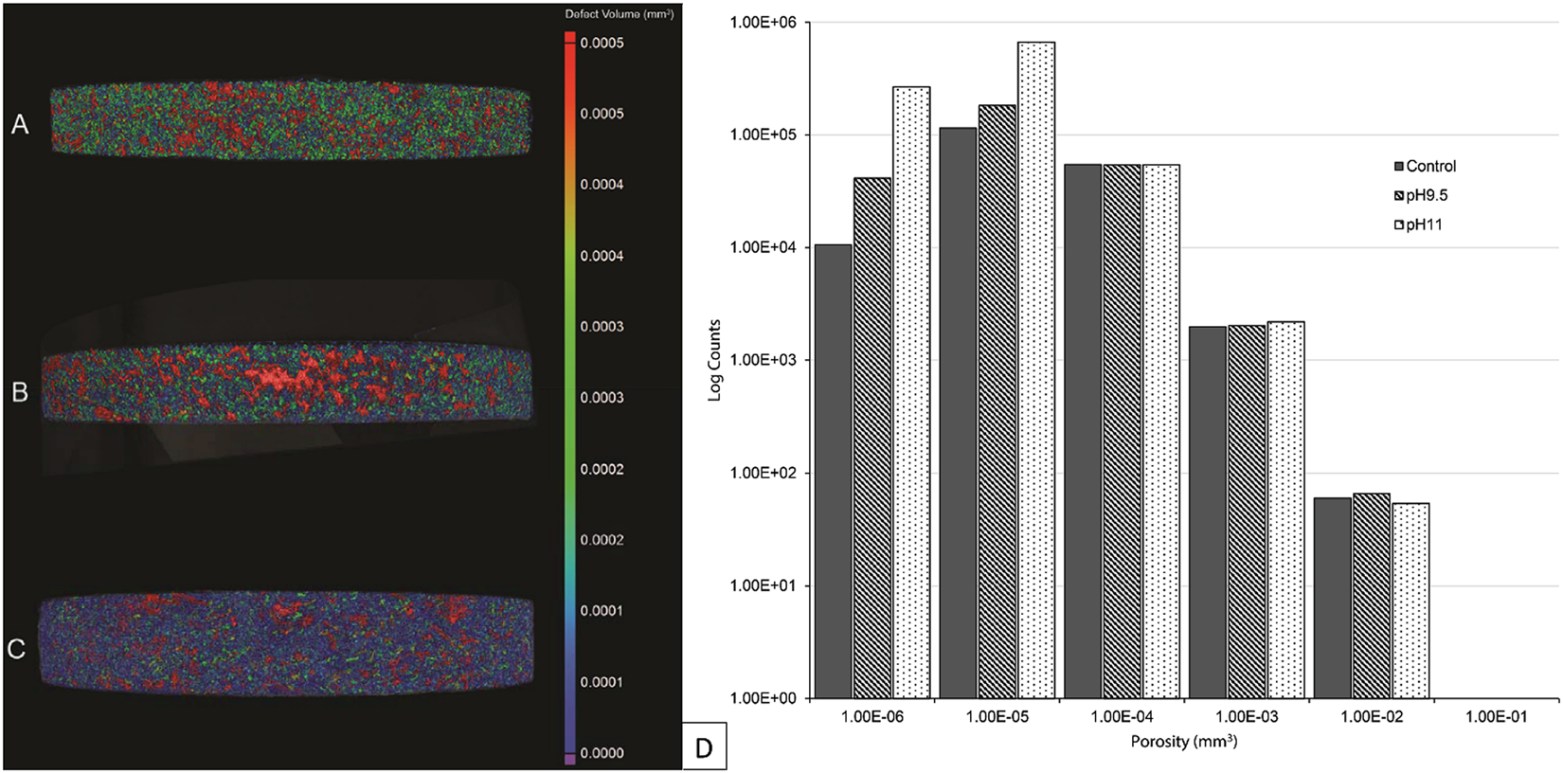
CT scanning of ^13^C doped simulants. (Left) The simulant surface prior to insertion to microcosms can be seen in (**A**), the pH 9.5 (**B**) and pH 11.0 (**C**) microcosms associated surfaces are also shown. The associated defect volumes and Log counts associated with those volumes of each of the surfaces is also shown (**D**).

Biofilms formed readily on the surfaces of the ^13^C simulants at both pH 9.5 and 11.0 (Fig. 2A and B), however MIMS analysis of the reactor fluid found no evidence of ^13^C mobilisation or the generation of ^13^C bearing gases (Fig. S3), indicating that if present they were at a concentration below the 1 µg/L limit of detection. Where simulants were incubated in sterile controls no surface modifications were observed. Milling of the samples via FIB-SEM (Fig. 2C and D) showed that the biofilm did not penetrate the ‘cauliflower’ like topology of the deposit or result in any obvious removal or damage to the deposit. Rather, the biofilms formed over the top and around the deposit adding a low porosity surface layer at both pH 9.5 (Fig. 3B) and pH 11.0 (Fig. 3C) to the graphite (Fig. 3A) with pores in the 1 × 10^−5^ to 1 × 10^−6^ mm^3^ range (Fig. 3D).

**Figure 4:**
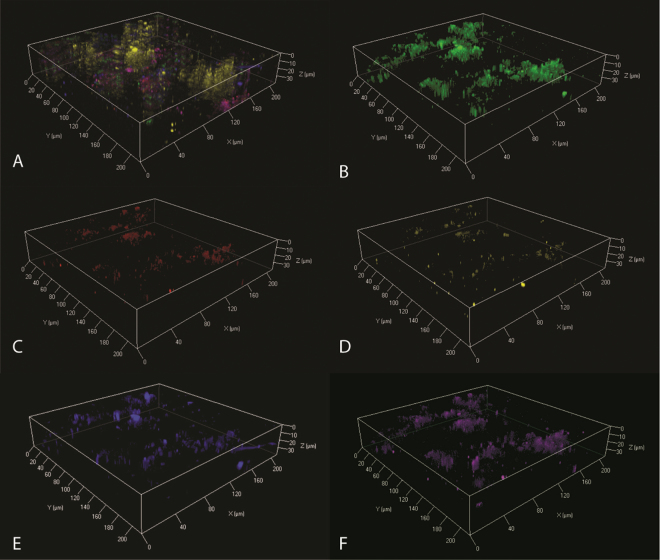
CLSM imaging of pH 9.5 C-13 simulant biofilm. Composite images of all stained components (**A**), comprising β-1,4 and β-1,3 polysaccharides (**B**), α-mannopyranosyl/α-glucopyranosyl sugars (**C**), lipids (**D**), proteins (**E**) and extracellular DNA (**F**).

**Figure 5:**
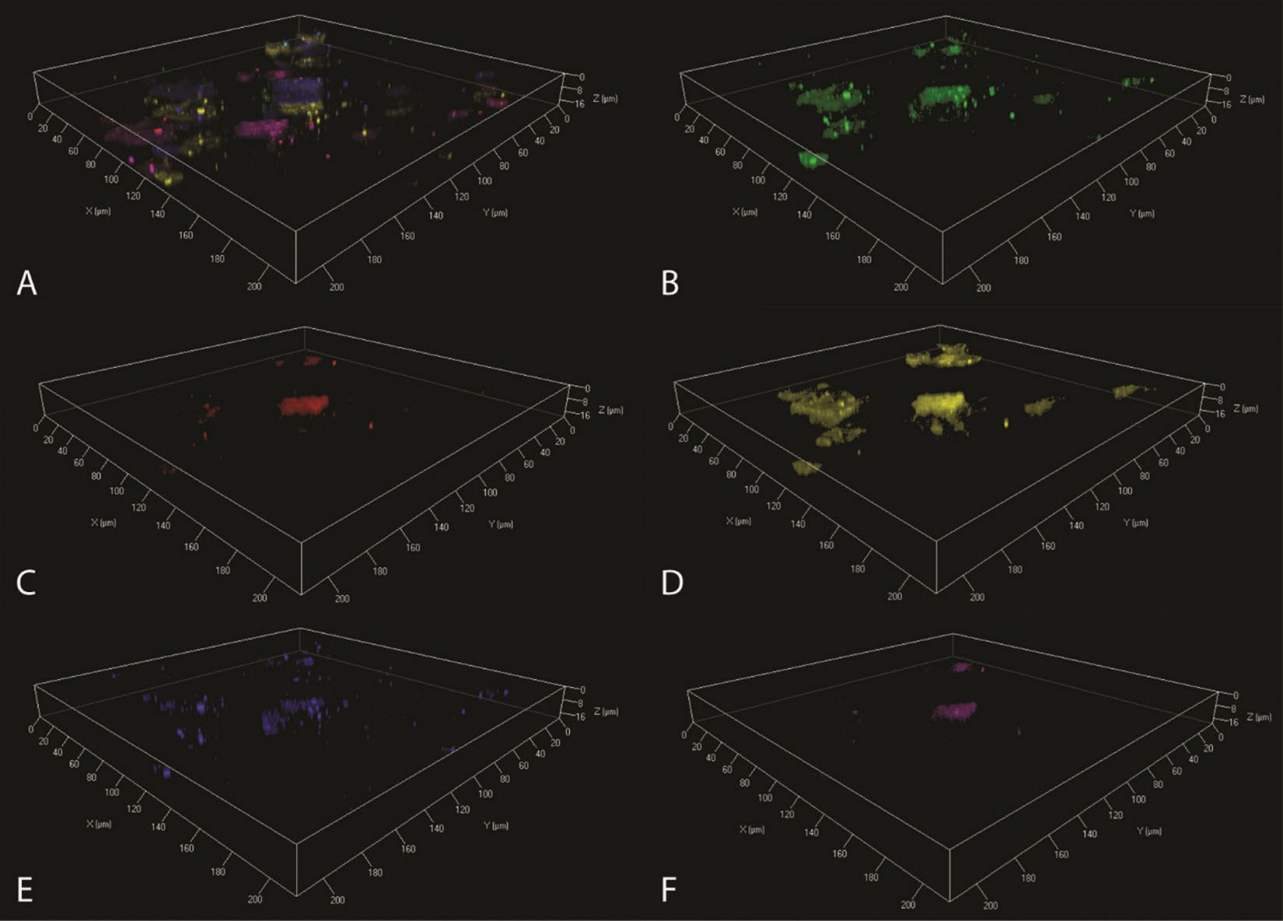
CLSM imaging of pH 11.0 C-13 simulant biofilm. Composite images of all stained components (**A**), comprising β-1,4 and β-1,3 polysaccharides (**B**), α-mannopyranosyl/α-glucopyranosyl sugars (**C**), lipids (**D**), proteins (**E**) and extracellular DNA (**F**).

The biofilm formed at pH 9.5 had significant amounts of extracellular DNA (eDNA) at the basal surface of the simulant (Fig. 4). The remaining structural elements were comprised of lipids, proteins, β-1,4 and β-1,3 polysaccharides and α-mannopyranosyl/α-glucopyranosyl sugars. The pH 11.0 biofilm also had an eDNA component at the surface of the simulant (Fig. 5), this was in turn covered by a clear lipid, β-1,4 and β-1,3 polysaccharides and α-mannopyranosyl/α-glucopyranosyl sugars layer (Fig. 5A,B,D and E) which was topped by a layer of proteins (Fig. 5A,C).

**Figure 6:**
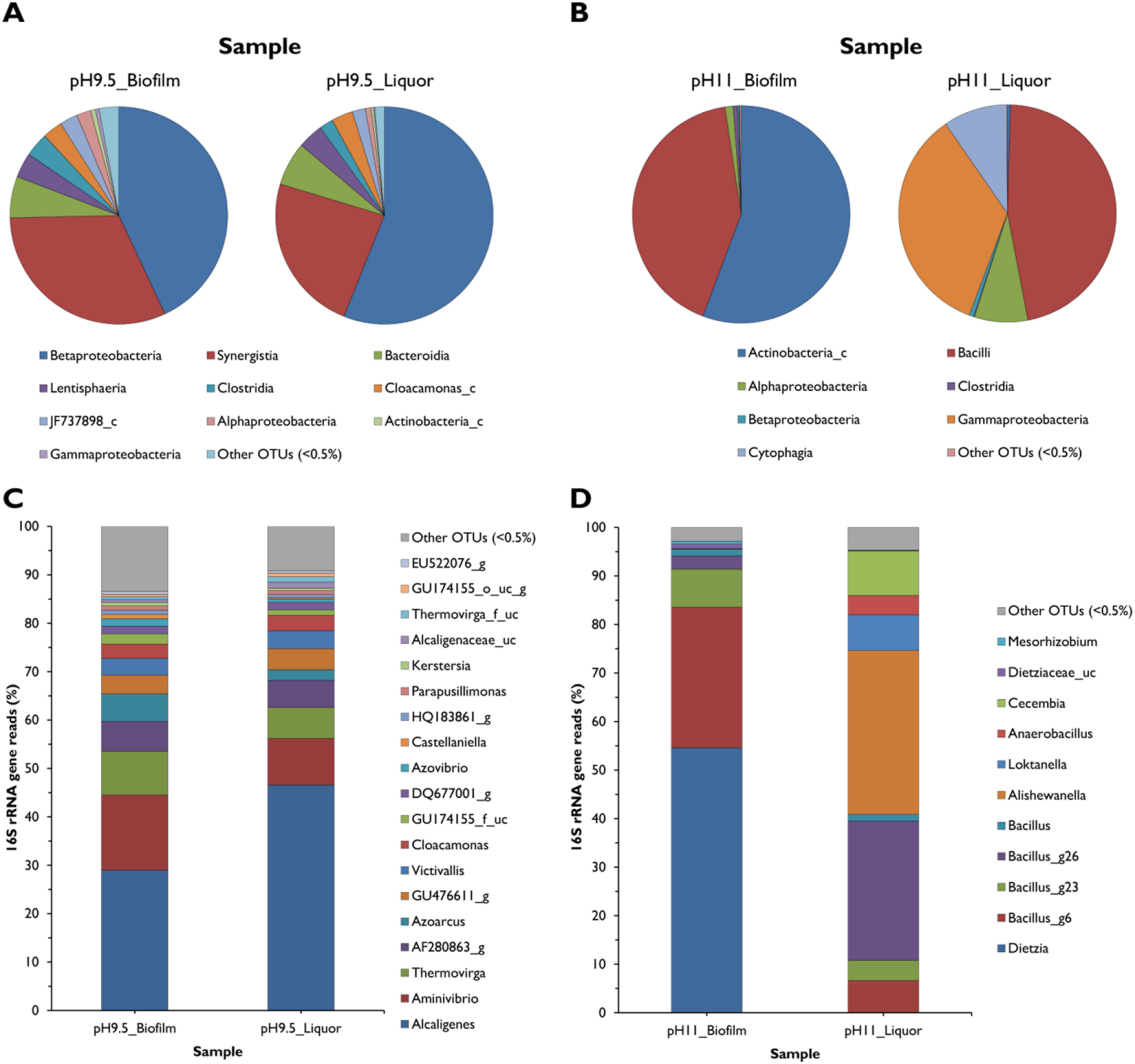
Miseq bacterial community profile. Comparisons of pH 9.5 (**A**) and pH 11.0 (**B**) reactor component profiles at the phylum classification, taxa at the species classification of the pH 9.5 (**C**) and pH 11.0 (**D**) reactor profiles.

Examination of the microbial populations present in the biofilms via principal co-ordinate analysis (Fig. S4) and quantitative comparison using a Cramer von Mises type statistic (Fig. S5) showed that there was a significant difference between the communities formed at each pH. The bacterial community analysis of the pH 9.5 communities can be seen in Fig. 6 (A, C and supporting material Fig. S6). Whilst the biofilm and liquor communities were similar the liquor community was more diverse in terms of the number of taxa observed. Both the biofilm and liquor communities were dominated by the Betaproteobacteria and Synergistia (75% of the biofilm and 80% liquor community) represented by *Alcaligenes sp* and a combination of *Aminivibrio* and *Thermovirga* respectively. Species of these genera have been observed to degrade amino acids under anaerobic conditions^24–26^.

At pH 11 the biofilm and liquor communities were more distinct (Fig. 6B,D). Within the biofilm, Actinobacteria and Bacilli accounted for ≈98% of the bacterial community with the majority of the Actinobacteria (98%) represented by *Dietzia sp* and Bacilli (Fig. 6D) showing homology to sequences of *Bacillus pseudofirmus* OF4. The liquor community was more diverse than the biofilm community where Bacilli (42%) and Gammaproteobacteria (35%) were dominant and the Cytophagia and Alphaproteobacteria (10% and 8% respectively) were secondary components. The Bacilli included reads showing similarity to the alkaliphilic *Bacillus nanhaiisediminis* strain NH3 (99.2% similarity) and species from the genus *Anaerobacillus* related to the alkaliphilic soda lake species, *Anaerobacillus alkalilacustris* strain Z-0521 (98.8%)^27^. At the genus level, the Gammaproteobacteria population was dominated by *Alishewanella* species of which have been previously observed within alkaliphilic flocculate communities capable of CDP degradation^16^.

In addition to bacteria, Archaea were also present within the community profiles of the samples obtained from the pH 9.5 liquor and biofilms (Fig. S6), but were not detected in the pH 11.0 communities. Both biofilm and liquor pH 9.5 Archaeal communities were dominated by species of the genus *Methanobacterium* showing greatest sequence homology to *Methanobacterium alcaliphilum* (99% sequence similarity) an alkaliphilic hydrogen oxidising methanogen^28^.

## Discussion

In the case of the radioactive samples, biofilms readily formed upon both fuel channel wall and inner brick surfaces. Indicating that the levels of radioactivity were not sufficient to prevent biofilm formation even though the ^14^C is concentrated in the surface deposit on the channel wall samples^4^. Bacterial interaction with the radioactive samples and metabolism of CDPs did not result in any detectable release or formation of ^14^C bearing species. This observation was also seen within the simulant associated microcosms where ^13^C bearing species (CH_4_ or CO_2_) were also undetectable even though the amount of ^13^C present was well within the detection limit of the MIMS system employed and significantly greater than the ^14^C content of the reactor graphite. It is therefore likely that microbial activity may not increase the mobility of the graphite associate ^14^C through the generation of ^14^C bearing gases, a mobilisation route considered within some modelling approaches^10^. In both the radioactive and simulant microcosms the cauliflower topology of the ^14/13^C surface deposits was evident, as was the fact that the biofilm matrix had partially covered these structures. Milling of the samples via a FIB indicated that the biofilms were not capable of penetrating these surface deposits. Rather microbial growth was limited to the surface, encasing the graphite in a layer of biofilm rather than causing any degradation to the graphite microstructure. The formation of this biofilm generated a layer of low porosity features on the surface on the graphite.

Although the two biofilms generated within this study showed a degree of variation in the polysaccharide composition, the lipids, proteins and eDNA content were consistent with previous observations of alkaliphilic systems^20^. The generation of eDNA is often associated with biofilm formation, in which it provides both a scaffold material as well as a potential source of nutrients^29,30^. In this study the eDNA formed the basal layer of the biofilm at both pH values, as previously observed at alkaline pH on virgin graphite^21^. This suggests that the simulated surface deposits investigated here did not alter biofilm initiation. The production of both lipids and proteins not only aid the structure and functional elements of the biofilm, they also contribute to protection from alkaline conditions by acting as a buffer via the production of acidic groups within the polymer^31^.

β-1,4 and β-1,3 polysaccharides were the major carbohydrate present within the pH 9.5 biofilm, with sparse detection of α-mannopyranosyl/α-glucopyranosyl sugars. In contrast, the opposite was observed within the pH 11.0 biofilm, which was dominated by these α-mannopyranosyl/α-glucopyranosyl sugars. Rather than being a response to increase in pH, these variations in carbohydrate content appear to be due to the variation in community structures observed. The pH 9.5 biofilm was dominated by Betaproteobacteria, and in particular taxa of the genus *Alcaligenes*. Taxa associated with the genus *Dietzia* dominated the pH 11.0 biofilm community. This dominance of the *Dietzia* was almost certainly reliant upon its existence within a biofilm; since a limited number of *Dietzia* associated reads were observed within the planktonic component of the community. This correlates with previous studies in which *Dietzia* exhibited enhanced survival in alkaline conditions within a biofilm structure^20,32^ indicating that this genus is a key component of alkaliphilic biofilms.

Principal co-ordinate analysis (Fig. S3) and quantitative comparison using a Cramer von Mises type statistic (Fig. S4) showed that there was a significant difference between the two biofilm communities. Despite this difference, a number of taxa within each community profile were capable of a range of metabolic activities which are likely to contribute to the degradation of CDP as they had done within planktonic reactions described previously^16,17^.

Overall the study represents a culmination of the integrated work packages of the C14-BIG project and demonstrates for the first time that polymicrobial communities are capable of forming complex biofilms on the surface of irradiated graphite under alkaline conditions. Our findings show that these biofilms form by utilising CDPs as a carbon source; which is likely to be present from other ILW packages. In addition, the ^13^C simulant developed as part of the project was used to provide a more extensive insight into the characteristics of an irradiated graphite associated biofilm. However, there was no evidence of any enhancement of the release of either ^14^C or ^13^C from the graphite samples investigated.

## Materials and Methods

### Irradiated graphite microcosms

Two previously described microcosms operating at pH 9.5^15^ and 11.0^16^ were chosen as seed cultures for biofilm formation. Sub-samples (90 ml) of the seed cultures were transferred to sterile, anoxic flasks containing single trepanned (12 mm Ø × 6 mm, 1 g in weight) irradiated graphite samples originating from inner brick or channel wall graphite from the Oldbury nuclear power station^22^. The pH of these reaction vessels was adjusted to pH 9.5 and pH 11.0 if necessary under nitrogen using 4 M NaOH following the addition of cellulose degradation products (CDP, 10 ml) as per Rout *et al*.^17^. Nitrogen flushed reaction vessels were then sealed and stored in an argon atmosphere for 12 weeks at 25 °C. Alongside these microbial samples, a set of control experiments were prepared in which active sample was incubated within mineral media used in the preparation of the microcosms^33^. At the end of incubation, headspace gas samples (2 ml) were removed using a syringe and hypodermic needle before being injected into a specialised gas rig containing a small quadrupole mass spectrometer (Fig. S1), known as a residual gas analyser (RGA). The use of a leak valve set at 3.0 × 10^−6^ mbar allowed for the evacuation of the vacuum cell prior to the injection of headspace gas samples. For baseline comparison an empty vessel within the glovebox was also sampled as a negative control. Liquid scintillation counting (LSC) using ScintLogic U scintillation cocktail (LabLogic, Sheffield, UK) was also employed by injecting a sample of headspace gas into an LSC vial modified to hold a septum. In addition, a sample (2 ml) of the liquid fraction was also removed and mixed with 2 ml of the same scintillation cocktail in a plastic scintillation vial. ^14^C bearing species analysis was then performed using a Hidex triathler LSC with a counting time of 60 minutes and a counting window of 20–200 keV to reject ^3^H associated counts. An un-amended scintillation vial was used as a negative control, the associated limits of detection were 6.8 Bq/ml for the gas analysis and 4.3 Bg/ml for the liquid analysis. The graphite sample was removed from the solution and examined in the variable pressure mode of a Zeiss Sigma SEM to allow for surface examination using backscatter electron imaging.

### ^13^C graphite Microcosms

^13^C labelled carbonaceous deposits were prepared on unirradiated Pile Grade A graphite discs (Ø12 mm × 1 mm) via microwave plasma chemical vapour deposition described in Payne *et al*.^23^. These discs held 3.6 mg of ^13^C, which equates to 0.012 mg/mm^2^. Three discs were incubated within a biofilm reactor (CDC reactor, Biosurface Technologies Corp., US.) in the presence of seed cultures and CDP to provide sufficient samples for SEM, CLSM, CT and community analysis. All reagents were flushed for 30 minutes with nitrogen prior to use and the inocula prepared under a stream of nitrogen. Simulants were then immersed within the suspension and the biofilm reactor sealed to maintain anoxic conditions. The reactor was stirred at 120 rpm and incubated at 25 °C, with 50 ml of the total volume removed and replaced with fresh cellulose degradation products every 2 weeks and pH attenuated to either pH 9.5 or pH 11.0 using 4 M NaOH. Each biofilm reactor was operated for 12 weeks. In addition, control reactors were prepared in which simulants were suspended in mineral media at pH 9.5 and pH 11.0. The presence of ^13^C-bearing gases within the aqueous phase was then determined using a membrane inlet mass spectrometer (MIMS) (Hiden Analytical Ltd, UK) which has a detection limit of 1 µg/L. Following incubation a portion of each biofilm was removed using a sterile scalpel blade and transferred to individual sterile 50 ml tube and fixed by submerging in 4% paraformaldehyde (Fisher, UK) overnight. A staining regimen was then performed in accordance with methods outlined in Chen *et al*.^34^. The fixed and stained biofilm was visualised via confocal laser scanning microscopy (CLSM) at the Bio-imaging centre of Leeds University using a Zeiss LSM880 inverted confocal microscope with image analysis performed using Zen 2.1 (Zeiss Microscopy).

### Porosity Measurements

Sample porosity data were captured via X-ray tomography (XCT) using a Nikon XTH 225 (Nikon Metrology, Tring, UK) fitted with a tungsten reflection target which has a maximum focal spot size of 3 µm and a complementary metal-oxide semiconductor (CMOS) 1000 × 1000 pixel flat panel detector. CT Pro (Nikon Metrology, Tring, UK) and VG Studio 2.2 (VGS) (Volume Graphics GmbH, Heidelberg, Germany) software packages were used to perform reconstruction and analysis. Samples were fixed vertically to the detector to prevent noise/scattering. Data were captured with 1583 total projections at 80 kV and 105 µA throughout, resulting in a voxel size of 15.56 μm. Projections were then reconstructed into volume datasets for each sample and defect detection based on voxel-size was performed to compare porosity levels between samples.

### Microscopy

The EPS composition and morphology of biofilm communities was investigated via CLSM using a Zeiss LSM880 inverted confocal microscope with image analysis performed using Zen 2.1 (Zeiss Microscopy). Samples of biofilm were stained using the following compounds in accordance with methods outlined in Chen *et al*.^34^: Calcofluor white for the visualisation of β-1,4 and β-1,3 polysaccharides (Sigma, UK), Nile red (Fisher, UK) for lipids and hydrophobic sites, Concanavalin A, Tetramethylrhodamine Conjugate (Fisher, UK) for α-Mannopyranosyl, α-glucopyranosyl sugars, FITc (Fisher, UK) for protein and Syto 63 (Fisher, UK) for total cells and extracellular DNA.

Electron microscopy was carried out using a Helios NanoLab 600i combined SEM/FIB system (FEI, Oregon USA). The focused ion beam (FIB) was utilised to precision mill trenches to allow the thickness and morphology of both the biofilm and the deposit to be determined. Electron micrographs were acquired using an accelerating voltage of 20 kV. Trenches were FIB milled with the use of a Ga + ion source with an accelerating voltage of 30 kV. The milled trenches had approximate dimensions of 50 μm × 56 μm × 20 μm (x, y and z respectively). After FIB milling a Carl Zeiss Sigma™ Variable Pressure Scanning Electron Microscope (SEM) with a Gemini™ field emission electron column was used to collect secondary electron and backscattered electron images from the surface and sub-surface of the samples. A consistent 25 kV accelerating voltage, 120 μm aperture and 2.3 A current were used throughout.

### 16S rRNA gene community analysis

At the end of operation, 50 ml of the liquor was removed from the cultures and centrifuged at 8,000 × g for 20 minutes and the pellet reconstituted with 4 ml of phosphate buffered saline (pH 7.0). In addition, a portion of the biofilm was removed from the disk using a fresh scalpel blade and rinsed with PBS to remove any transient organisms from the liquor. DNA was then extracted from both the liquor and biofilm using a Powersoil DNA extraction Kit (Mo-Bio, US), with the homogenisation/lysis step increased to 45 minutes when extracting from biofilm. The V4 region of the 16S rRNA gene was amplified using duel primers 519 F (5′CAGCMGCCGCGGTAA3′) and 785 R (5′TACNVGGGTATCTAATCC3′) for both bacteria and archaea^35,36^ with the following overhangs 5′TCGTCGGCAGCGTCAGATGTGTATAAGAGACAG3′ and 5′GTCTCGTGGGCTCGGAGATGTGTATAAGAGACAG3′, respectively. PCR products were purified using a Qiaquick PCR purification kit (Qiagen, UK) and 16S microbial Community analysis was carried out via a MiSeq platform (Illumina, USA) at 250 bp paired ends with chimera detection and removal performed via the UNCHIME algorithm in the Mothur suite^37^ (Chunlab, South Korea) before identification using the EzTaxon-e database^38^ (http://eztaxon-e.ezbiocloud.net/). Comparative statistical analysis of the taxa observed within the communities were carried out via a Cramér von Mises-type statistic followed by a Monte Carlo test procedure as described by Singleton *et al*.^39^. Miseq sequencing data are available within NCBI GenBank BioProject (https://www.ncbi.nlm.nih.gov/bioproject/) PRJNA314287 under SRA: SAMN04530554, SAMN04530555, SAMN04530556 and SAMN04530557.

## Electronic supplementary material

Supporting information
