# Supplementary material for: The Impact of Alkaliphilic Biofilm Formation on the Release and Retention of Carbon Isotopes from Nuclear Reactor Graphite
Source: Sci Rep. 2018 Mar 13;8:4455. doi: 10.1038/s41598-018-22833-5 (PMC5849744; doi:10.1038/s41598-018-22833-5)
Supplement: Supplementary file 1 — Supporting information [file 41598_2018_22833_MOESM1_ESM.docx]

# Supporting information

The Impact of Alkaliphilic Biofilm Formation on the Release and Retention of Carbon Isotopes from Nuclear Reactor Graphite.

S. P. Rout^1^, L. Payne^2^, S. Walker^3^, T Scott^2^, P. Heard^2^, H. Eccles^4^, G. Bond^3^, P. Shah^5^, P. Bills^5^, B. R. Jackson^6^, S. A. Boxall^6^, A. P. Laws^7^, C. Charles^1^, [S. J. Williams](http://www.sciencedirect.com/science/article/pii/S0022311515303901)^8^ and P. N. Humphreys^1∗^


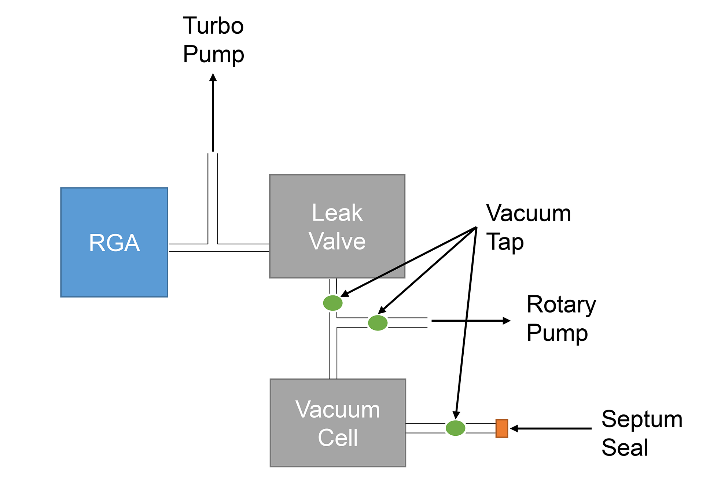


**Figure S1: Schematic of RGA rig**

**Figure S2: LSC of the liquid and headspace gas phases**.

**Figure S3: MIMS analysis of simulant microcosm**

**
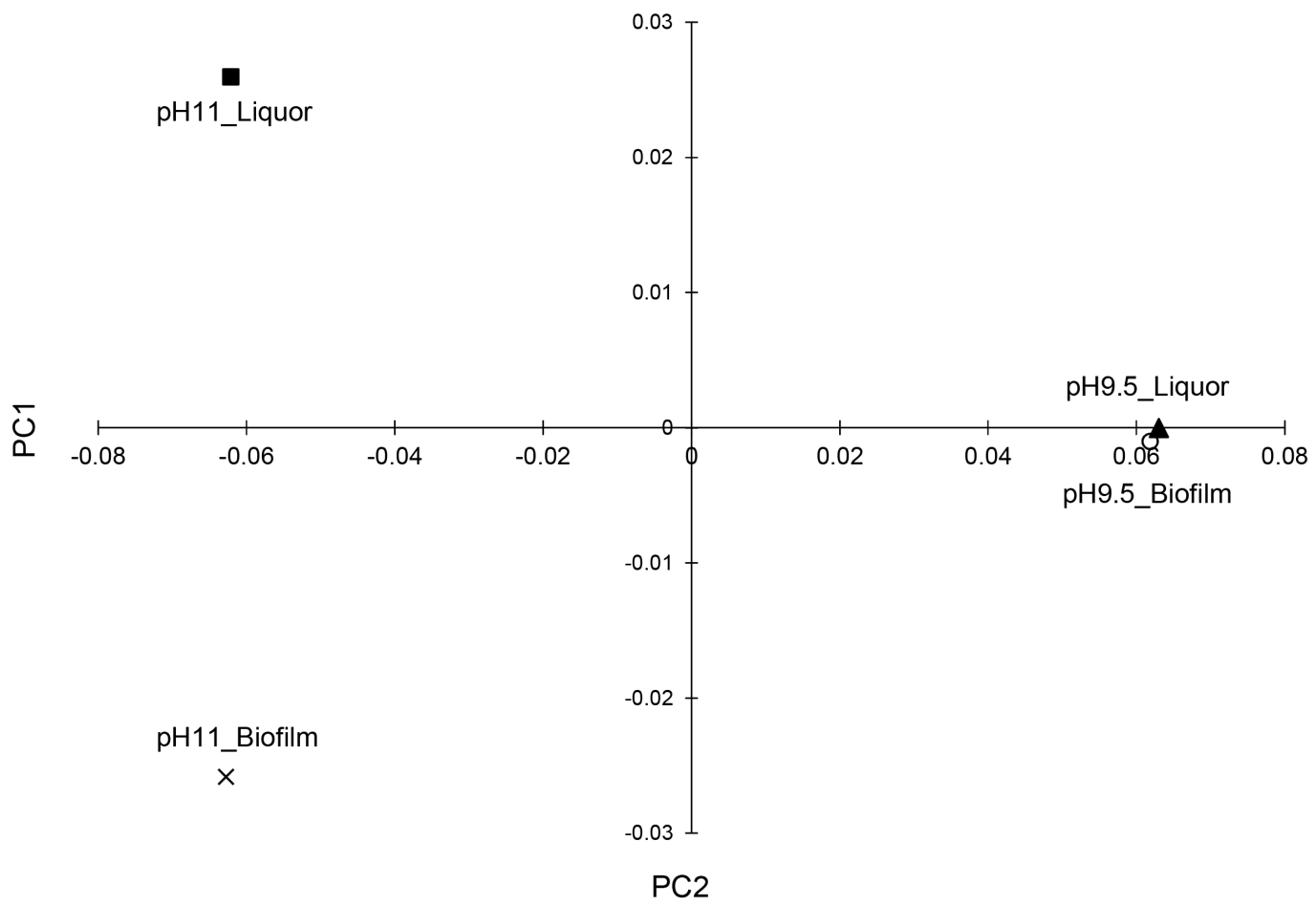
**

**Figure S4: Principal Co-ordinate analysis of Miseq sample libraries**


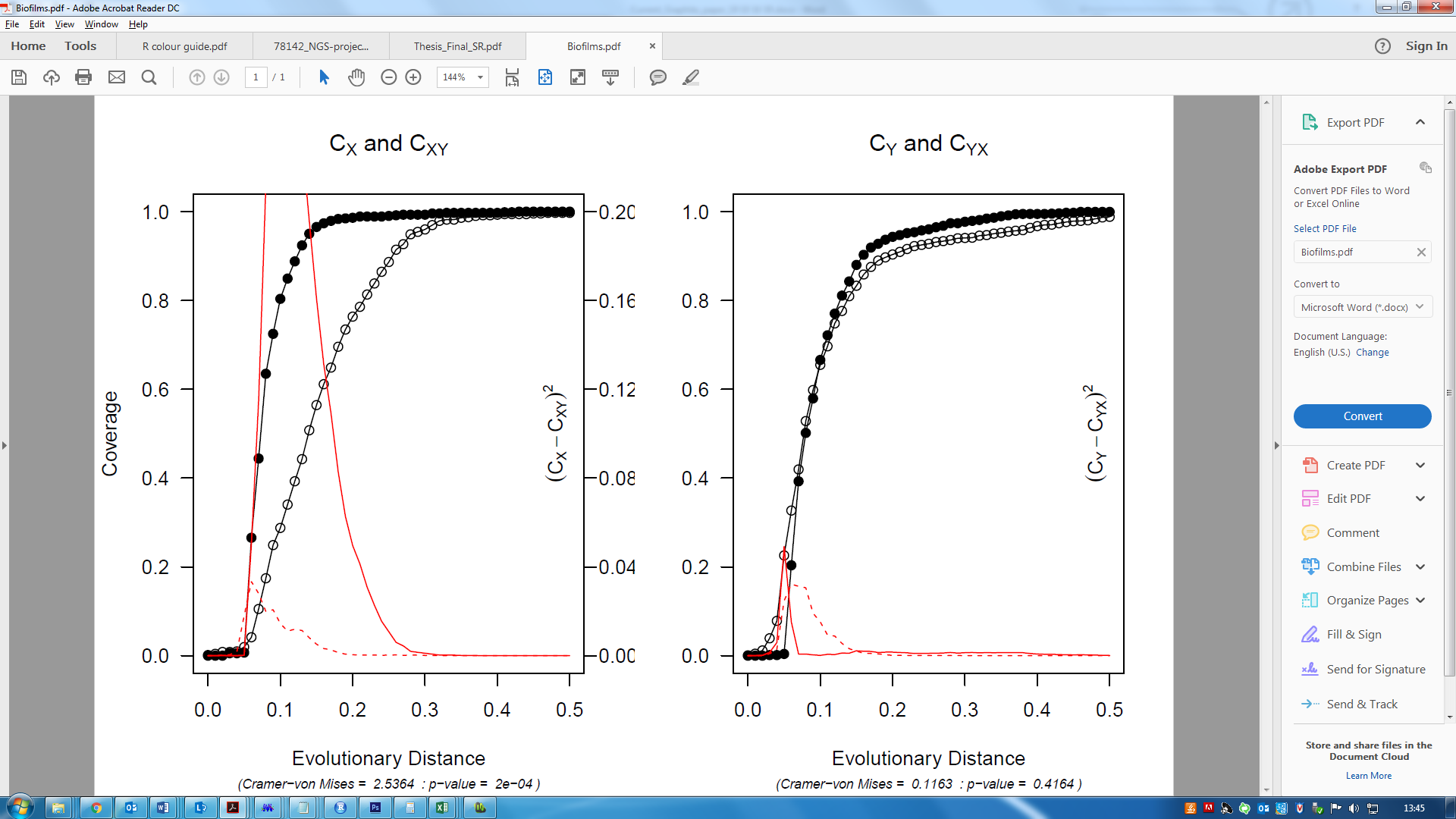


**Figure S5: Cramer von Mises-type statistical analysis of biofilm communities.**

**Figure S6: Community composition of Archaea associated 16S rRNA gene reads.**
